# The Influence of Dietary Factors on Melanoma Development and Progression: A Comprehensive Review

**DOI:** 10.3390/nu17111891

**Published:** 2025-05-31

**Authors:** Abigail E. Watson, Nabiha Yusuf

**Affiliations:** 1 College of Medicine, Florida State University, Tallahassee, FL 32306, USA; 2 Department of Dermatology, University of Alabama at Birmingham, Birmingham, AL 35294, USA

**Keywords:** melanoma, diet, oxidative stress, inflammation

## Abstract

Melanoma is an aggressive cutaneous malignancy with increasing global incidence and high metastatic potential. While ultraviolet (UV) radiation remains the primary environmental risk factor, emerging evidence suggests that dietary factors may influence melanoma risk, progression, and treatment outcomes. This comprehensive review examines the impact of dietary components, including fats, vitamins, minerals, antioxidants, bioactive compounds, and the gut microbiome, on melanoma pathogenesis. The current literature indicates that diets rich in polyunsaturated fatty acids (PUFAs), antioxidants, and plant-based bioactive compounds may confer protective effects against melanoma by modulating oxidative stress, inflammation, and immune response. Additionally, the gut microbiome plays a critical role in melanoma progression and immunotherapy response, with dietary patterns influencing microbial composition and, consequently, host immunity. Despite these promising associations, research remains limited, and findings across studies are inconsistent, preventing the establishment of definitive dietary guidelines for melanoma prevention and management. Future research should focus on large-scale prospective studies to elucidate the mechanisms underlying the dietary influences on melanoma and determine evidence-based nutritional strategies. Understanding the interplay between diet, immune modulation, and gut microbiome composition represents a promising avenue for advancing melanoma prevention and treatment strategies.

## 1. Introduction

Cutaneous melanoma is a malignant neoplasm arising from melanocytes, the pigment-producing cells in the skin. The pathogenesis of melanoma is multifactorial, with well-established risk factors including UV radiation exposure, the presence of melanocytic nevi, family history, and personal history of melanoma. In the recent literature, gastrointestinal microbiota have emerged as a potential environmental factor influencing melanoma development and progression [1]. This diverse microbial community, comprising approximately 1000 species of commensal and symbiotic microorganisms, including bacteria, archaea, viruses, and unicellular eukaryotes, has been well-documented as modulating inflammation, immune evasion, and carcinogenesis [1]. The disruption of the gastrointestinal microbial ecosystem may contribute to a pro-inflammatory state, potentially influencing melanoma pathogenesis.

Globally, the incidence of cutaneous melanoma continues to rise, with intermittent high-intensity UV radiation exposure recognized as a major environmental risk factor [2]. While the genetic and environmental factors contributing to melanoma are well-established, there is growing interest in the potential role of dietary factors in modifying cancer risk and outcomes. Dietary modifications have been associated with a reduced risk of other malignancies, such as colorectal and breast cancer. However, evidence linking diet to melanoma development and progression remains limited. Patients and caregivers often seek dietary guidance to optimize cancer outcomes, but the lack of clinical data specific to melanoma presents a challenge for providers in providing evidence-based dietary recommendations. This review aims to evaluate the literature regarding the influence of dietary factors on melanoma development and progression. By reviewing the existing evidence, we aim to identify potential dietary interventions that may contribute to improved melanoma outcomes and inform future research directions.

## 2. Dietary Influence on Melanoma Pathogenesis

### 2.1. Oxidative Stress and Antioxidants

Oxidative stress, defined as an imbalance between the production of reactive oxygen species (ROS) and the capacity of the antioxidant defense system, plays a critical role in melanoma pathogenesis and progression [3]. During melanin biosynthesis, melanocytes produce significant levels of hydrogen peroxide (H_2_O_2_) while simultaneously depleting reduced glutathione (GSH), a critical antioxidant responsible for neutralizing ROS [4]. This intrinsic pro-oxidant state is further exacerbated by UV radiation exposure, which not only induces direct DNA damage but also promotes excessive ROS formation, compounding oxidative stress within melanocytes [4]. Given this biological vulnerability, strategies aimed at reducing oxidative stress may have therapeutic potential in mitigating the development and progression of melanoma. A key factor influencing oxidative stress burden in melanocytes is the type of melanin produced. Pheomelanin, the lighter pigment more prevalent in individuals with fair skin, has been shown to promote ROS generation upon UV exposure [5]. Conversely, eumelanin, the darker pigment dominant in individuals with darker skin tones, possesses antioxidative properties that reduce ROS production and offer protective effects against UV-induced damage [5]. Consequently, individuals with higher pheomelanin-to-eumelanin ratios face an increased oxidative burden and a higher risk of melanoma development, largely due to chronic oxidative stress [5]. This mechanistic link underscores the importance of modulating oxidative stress as a potential approach to reducing melanoma risk. In addition to promoting melanoma initiation, oxidative stress plays a significant role in disease progression. Excessive ROS disrupt cellular homeostasis, promote genomic instability, and activate pro-carcinogenic signaling pathways, ultimately enhancing tumor growth and metastatic potential [6]. Moreover, oxidative stress can alter ion channel activity, which modulates cell proliferation, migration, and apoptosis, key factors influencing melanoma progression [6]. Studies have also demonstrated that chronic oxidative stress promotes systemic inflammation, immune suppression, and angiogenesis, further exacerbating tumor aggressiveness.

Melanocytes are inherently prone to oxidative damage due to their constant exposure to ultraviolet A (UVA) radiation and the oxidative byproducts generated during melanin synthesis [7]. This biosynthetic process involves oxidation reactions that produce ROS, such as superoxide anion and H_2_O_2_, contributing to a high oxidative burden within melanocytes. Studies have demonstrated that melanoma cells exhibit an altered redox homeostasis characterized by elevated intracellular ROS levels and impaired antioxidant defenses. In melanocytes isolated from melanoma patients, increased oxidative sensitivity has been linked to an imbalance in antioxidant mechanisms and the aberrant activation of redox-sensitive transcription factors like AP-1 and NF-κB [8]. Notably, in metastatic melanoma, genes responsible for antioxidant responses are frequently downregulated, further exacerbating ROS accumulation and promoting dedifferentiation and metastatic potential [9]. In contrast, some of these antioxidant genes are upregulated in non-metastatic cells, suggesting a stage-specific adaptation to oxidative stress during melanoma progression [9].

Histological analyses of human melanoma tissues have revealed elevated levels of oxidative damage markers, such as malondialdehyde (MDA), alongside the upregulation of antioxidant enzymes compared to benign naevi and surrounding non-tumorous skin [10]. Interestingly, MDA accumulation was not limited to melanoma cells but was also present in adjacent keratinocytes, indicating a broader impact of oxidative stress in the tumor microenvironment. Unlike non-melanoma skin cancers, which often show reduced antioxidant capacity, melanoma appears to selectively enhance certain antioxidant defenses, possibly as a survival mechanism in response to ongoing oxidative pressure [11]. GSH, a key cellular antioxidant, has also been implicated in melanoma metastasis. In murine models, highly metastatic B16M melanoma cells utilize GSH to resist oxidative and nitrosative stress within the liver vasculature [12]. Enhancing GSH levels in these cells promotes metastatic growth, while exposure to reactive nitrogen species such as nitric oxide can impair antioxidant enzyme activity, notably γ-glutamylcysteine synthetase (γ-GCS), further modulating redox regulation in the tumor environment [12].

The recent literature also suggests that dietary factors may influence the oxidative stress burden within the body, potentially impacting melanoma risk and progression. Diets rich in antioxidants, such as vitamins C, E, and polyphenols, have been shown to reduce oxidative stress by neutralizing ROS, thereby protecting cells from oxidative damage [13]. Additionally, dietary intake of omega-3 fatty acids has been demonstrated to have anti-inflammatory and antioxidative effects, which may counteract the pro-inflammatory microenvironment that fosters melanoma progression [14]. Conversely, diets high in processed meats, refined carbohydrates, and saturated fats have been associated with increased oxidative stress and inflammation, potentially accelerating tumor progression. Given the central role of oxidative stress in melanoma pathogenesis, dietary interventions aimed at reducing oxidative stress may offer a promising approach for modifying melanoma risk and outcomes. Biomarker studies in melanoma patients further support the link between oxidative stress and disease progression, with notable alterations in oxidative stress markers observed in advanced-stage melanoma. Elevated MDA levels, a marker of lipid peroxidation and oxidative stress, have been consistently reported in melanoma patients, particularly in those with metastatic disease [15]. Moreover, changes in antioxidant enzyme activity have been shown to correlate with melanoma stage. For instance, superoxide dismutase (SOD) activity is significantly elevated in stage III melanoma, while SOD2 activity predominantly increases in stage IV melanoma [15]. Additionally, catalase activity, another critical antioxidant enzyme, appears to decline in stage IV melanoma, suggesting the progressive depletion of the body’s antioxidant defenses in advanced disease [4].

Despite the growing recognition of oxidative stress as a driver of melanoma pathogenesis, dietary interventions targeting oxidative stress remain underexplored in melanoma research. While extensive evidence supports the protective role of antioxidant-rich diets in reducing the risk of other malignancies, such as colorectal and breast cancer, data linking dietary factors to melanoma prevention or progression remain limited. However, given the clear mechanistic link between oxidative stress and melanoma development, future research exploring the impact of diet on oxidative stress modulation in melanoma is warranted. Understanding how dietary patterns, nutrient intake, and bioactive compounds influence redox homeostasis may offer novel preventative and therapeutic strategies to mitigate melanoma risk and improve patient outcomes.

### 2.2. Inflammation

Chronic inflammation plays a critical role in melanoma development and progression by fostering an immunosuppressive tumor microenvironment that supports tumor growth, invasion, and immune evasion. Inflammatory cells, such as macrophages, regulatory T cells (Tregs), and myeloid-derived suppressor cells (MDSCs), infiltrate the tumor site and secrete immunosuppressive molecules, including transforming growth factor-beta (TGF-β), interleukin-10 (IL-10), vascular endothelial growth factor (VEGF), and prostaglandin E2 (PGE2) [16,17]. These cytokines contribute to the expansion and activation of Tregs, which subsequently inhibit cytotoxic CD4+/CD8+ T cell proliferation and function, diminishing the immune system’s capacity to control tumor growth [17]. Additionally, macrophages at the tumor site produce indoleamine 2,3-dioxygenase (IDO), an immunosuppressive enzyme that depletes tryptophan, a critical amino acid required for T cell activation [18]. This process not only suppresses T cell proliferation but also enhances the recruitment of Tregs, further promoting immune evasion and melanoma progression. The inflammatory tumor microenvironment is also sustained through metabolic dysregulation, oxidative stress, and immune suppression. The increased secretion of pro-inflammatory cytokines promotes a state of chronic low-grade inflammation, which has been associated with more aggressive melanoma phenotypes and poorer clinical outcomes. Biomarker studies have demonstrated that abnormal serum levels of IDO, lactate dehydrogenase (LDH), and S100B correlate with advanced melanoma stages and poorer prognosis [19]. Specifically, decreased IDO levels in melanoma patients are associated with shorter overall survival, increased recurrence rates, and accelerated tumor progression, further emphasizing the critical role of inflammation in driving melanoma pathogenesis [19,20].

Growing evidence suggests that diet significantly influences systemic inflammation and immune function, presenting a potential modifiable factor in melanoma prevention and management. Excessive adipose tissue, commonly associated with diets high in saturated fats, processed foods, and refined sugars, has been shown to induce a chronic inflammatory state through the secretion of pro-inflammatory adipokines, increased insulin resistance, and elevated levels of insulin-like growth factor-1 (IGF-1) [21]. These factors collectively promote systemic inflammation, creating a microenvironment favorable for tumor growth and immune suppression. Additionally, elevated circulating levels of pro-inflammatory cytokines, such as tumor necrosis factor-alpha (TNF-α), interleukin (IL)-6, and C-reactive protein (CRP), have been linked to increased melanoma progression and poorer clinical outcomes [22]. Conversely, dietary patterns characterized by a high intake of anti-inflammatory and immunomodulatory foods have demonstrated the potential to counteract chronic inflammation, potentially mitigating melanoma risk and progression. Diets rich in fruits, vegetables, whole grains, nuts, seeds, and omega-3 fatty acids have been shown to downregulate pro-inflammatory cytokine production, enhance antioxidant defenses, and improve immune function [14]. Polyphenols, flavonoids, and carotenoids commonly found in plant-based diets exhibit potent anti-inflammatory properties by inhibiting nuclear factor kappa-light-chain-enhancer of activated B cells (NF-κB) and reducing the production of inflammatory cytokines. Moreover, omega-3 fatty acids, primarily found in fatty fish, exert immunomodulatory effects by promoting the synthesis of anti-inflammatory eicosanoids, which may help restore immune surveillance and reduce tumor-promoting inflammation [13].

The relationship between dietary patterns and melanoma progression is further supported by clinical data demonstrating a correlation between obesity, chronic inflammation, and melanoma outcomes. Excessive adipose tissue not only acts as a reservoir for pro-inflammatory cytokines but also promotes the proliferation of immunosuppressive Tregs, impairs dendritic cell (DC) function, and facilitates the expansion of MDSCs, collectively undermining antitumor immune responses [23]. Furthermore, diets high in sugar and refined carbohydrates exacerbate systemic inflammation through hyperinsulinemia and the increased production of IGF-1, both of which have been associated with enhanced tumor cell proliferation and metastasis in melanoma [23]. Although there is growing evidence connecting chronic inflammation to melanoma development, dietary strategies aimed at mitigating this risk remain largely unexplored. However, given the well-documented anti-inflammatory and immunomodulatory effects of certain nutrients, diet may offer a promising means of reducing inflammation and potentially influencing melanoma progression.

### 2.3. Gut Microbiome Modulation

The gut microbiome, consisting of bacteria, fungi, viruses, protozoa, and bacteriophages, plays a critical role in shaping immune homeostasis and influencing cancer progression, including melanoma. It is estimated that the human body harbors approximately 4 × 10^13^ microbial cells, with over 95% residing in the gastrointestinal tract [24]. This vast microbial community profoundly impacts local and systemic immune responses, contributing to immune tolerance, inflammation, and antitumor immunity. Increasing evidence suggests that gut dysbiosis, defined as an imbalance in the gut’s microbial composition, may influence cancer development, progression, and response to immunotherapy, making the gut microbiome a significant and modifiable factor in melanoma outcomes.

A key mechanism through which the gut microbiome regulates immune function is through the production of bacterial fermentation products, such as short-chain fatty acids (SCFAs). SCFAs, including acetate, propionate, and butyrate, promote the expansion and function of regulatory Tregs in the intestine, facilitating immune tolerance and reducing inflammation [25]. Additionally, SCFAs inhibit histone deacetylases (HDACs), resulting in the hyperacetylation of histones within immune cells and subsequent downregulation of pro-inflammatory cytokines [26]. This immunomodulatory effect contributes to immune homeostasis but may also suppress antitumor immune responses in melanoma, allowing tumors to evade immune surveillance. Beyond SCFAs, certain bacterial species within the gut microbiome can drive systemic immunosuppression through distinct molecular pathways. For example, Bacteroides fragilis, a commensal bacterial species, produces polysaccharide A, which promotes the differentiation and expansion of IL-10-producing Tregs [27]. This immunosuppressive shift suppresses cytotoxic CD4+ and CD8+ T cell activity, reducing the immune system’s ability to mount an effective antitumor response against melanoma. Additionally, gut microbiota can promote systemic inflammation under dysbiotic conditions. Unmethylated cytosine phosphate guanosine (CpG) motifs in prokaryotic DNA, abundant in gut bacteria, activate toll-like receptor 9 (TLR9) on CD4+ and CD8+ T cells, promoting the release of pro-inflammatory cytokines such as interferon-gamma (IFN-γ) and IL-17 [28]. While inflammation is typically viewed as a tumor-promoting factor, in some cases, pro-inflammatory signaling can enhance immune surveillance against melanoma cells, complicating the role of the gut microbiome in melanoma progression.

Experimental models further illustrate the profound impact of the gut microbiome on systemic immune function and cancer progression. Germ-free (GF) mice, which lack intestinal microbiota, exhibit significantly impaired immune responses, including reduced numbers of DCs, a decrease in the phagocytic function of macrophages and neutrophils, and elevated circulating mast cells [29]. These immune deficiencies collectively impair antitumor immunity and increase melanoma susceptibility in GF mice, suggesting that a balanced gut microbiome is crucial for optimal immune function in melanoma defense. Furthermore, preclinical and clinical studies have demonstrated that alterations in gut microbiota composition can impact the response to immunotherapy in melanoma patients. For instance, gut microbiome profiles enriched with *Faecalibacterium* and *Ruminococcaceae* have been associated with favorable responses to immune checkpoint inhibitors (ICIs), whereas microbiomes dominated by Bacteroides species have been linked to poorer treatment outcomes [30]. This suggests that gut microbiota composition can modulate immunotherapy efficacy in melanoma and may serve as a therapeutic target to optimize treatment responses.

## 3. Nutrients and Food

### 3.1. Fruit and Vegetable Consumption

A diet rich in fruits and vegetables has been consistently linked to a lower risk of various cancers, including melanoma. Epidemiological studies suggest that individuals with a higher fruit and vegetable intake tend to have lower cancer rates and better overall health outcomes [31]. This protective effect is largely attributed to bioactive compounds such as polyphenols, flavonoids, carotenoids, and antioxidants, which help reduce oxidative stress, regulate inflammation, and support immune function. Polyphenols, found in berries, green tea, and citrus fruits, have demonstrated anti-inflammatory and immunomodulatory effects that may help prevent melanoma [13]. By inhibiting pro-inflammatory cytokines like tumor necrosis factor (TNF)-α and IL-6, these compounds may reduce the chronic inflammation that fuels tumor growth. Similarly, carotenoids such as beta-carotene—abundant in carrots, sweet potatoes, and leafy greens—act as powerful antioxidants, neutralizing ROS and protecting cells from UV-induced DNA damage [32]. Since oxidative stress is a key driver of melanoma, these antioxidant properties may offer an added layer of protection. Dietary fiber from fruits, vegetables, and whole grains also plays a role in melanoma outcomes by supporting gut health. A high-fiber diet promotes beneficial gut bacteria, leading to an increase in the production of SCFAs like butyrate, which helps regulate immune responses and reduce systemic inflammation. SCFAs may also enhance the effectiveness of immunotherapy in melanoma patients, suggesting that a diet rich in fruits and vegetables could be beneficial for both prevention and treatment [33].

### 3.2. Fats and Fatty Acids

The type and quantity of dietary fat intake may also play a significant role in melanoma development and progression. While total fat consumption has demonstrated conflicting associations with melanoma risk, the composition of dietary fat—particularly the ratio of omega-6 to omega-3 polyunsaturated fatty acids (PUFAs)—appears to have a more direct influence on melanoma outcomes [34]. Omega-6 PUFAs, predominantly found in vegetable oils, processed foods, and red meat, have been linked to increased pro-inflammatory signaling, tumor progression, and immune suppression in various cancers, including melanoma [35]. These pro-inflammatory effects are largely mediated by the arachidonic acid (AA) pathway, where omega-6 PUFAs promote the production of pro-inflammatory eicosanoids such as prostaglandins and leukotrienes, contributing to a microenvironment favorable for tumor growth.

Omega-3 PUFAs, particularly DHA, have garnered attention for their anticancer properties, which extend beyond general inflammation reduction and include the specific modulation of the tumor microenvironment and immune response. Epidemiologic data consistently demonstrate an inverse correlation between dietary omega-3 intake and cancer risk, and preclinical models have confirmed that DHA can suppress tumor proliferation, invasion, and metastasis while prolonging survival in tumor-bearing animals [36]. The mechanisms underlying these effects are multifaceted. Omega-3 PUFAs interfere with pro-carcinogenic signaling pathways, including through the inhibition of the NF-κB–COX2/PGE2 axis, downregulation of the SREBP-1c-FASN lipogenesis pathway, and attenuation of Akt-mTOR signaling—all of which are critical for tumor cell growth and survival [37]. Moreover, DHA enhances oxidative stress and lipid peroxidation within tumor cells, promoting apoptosis and ferroptosis. Its integration into cellular membranes also alters membrane fluidity and receptor dynamics, potentially disrupting pro-survival signaling cascades.

From an immunological perspective, omega-3 PUFAs exert profound effects on immune regulation, particularly through modulation of the cyclooxygenase (COX) and lipoxygenase (LOX) pathways [38]. Omega-3s compete with omega-6 fatty acids for COX enzyme binding, effectively reducing the synthesis of pro-inflammatory mediators such as PGE2. Elevated PGE2 levels, something common in diets high in omega-6 fatty acids, are associated with immunosuppression and have been linked to decreased tumor immunosurveillance and increased UV-induced carcinogenesis [38]. In contrast, omega-3 supplementation reduces PGE2 levels up to sevenfold compared to an equivalent omega-6 intake, thereby mitigating immune suppression and improving T-cell-mediated tumor rejection in models of UV-induced skin cancer [39].

T-cell metabolism is another key area influenced by fatty acid dynamics in the tumor microenvironment. CD8+ cytotoxic T lymphocytes (CTLs), essential for antitumor immunity, require robust metabolic support to sustain effector functions such as perforin and granzyme release [40]. Under conditions of nutrient depletion and hypoxia within the TME, CD8+ T cells increasingly rely on fatty acid oxidation for energy [41]. While this adaptation can support memory T cell development, excessive lipid uptake, particularly through CD36, a fatty acid transporter, has been shown to impair CTL function by promoting lipid peroxidation and ferroptosis, leading to functional exhaustion [41]. Notably, an increased expression of CD36 has been observed in melanoma and is associated with immune evasion. By reducing the availability of oxidizable lipids and limiting CD36-mediated uptake, omega-3 PUFAs may preserve CD8+ T cell function and bolster the antitumor immune response [42]. Additionally, omega-3s may indirectly modulate metabolic checkpoints such as the PI3K-Akt-mTOR axis and c-Myc, which are critical for the survival, proliferation, and differentiation of T cells [42].

Conversely, omega-3 PUFAs, commonly found in fatty fish, flaxseeds, and walnuts, have demonstrated anti-inflammatory and anti-tumorigenic effects. Omega-3 fatty acids, particularly eicosapentaenoic acid (EPA) and docosahexaenoic acid (DHA), inhibit the production of pro-inflammatory eicosanoids and promote the synthesis of anti-inflammatory mediators such as resolvins and protectins [39]. Preclinical studies have shown that increased omega-3 intake suppresses melanoma cell proliferation, reduces metastatic potential, and enhances immune function [39]. Moreover, a higher dietary intake of omega-3 PUFAs has been associated with improved responses to ICIs in melanoma patients, suggesting a potential role for dietary fat modulation as an adjunctive strategy in melanoma treatment.

### 3.3. Vitamins and Minerals

Vitamins and minerals also play a critical role in modulating melanoma risk and progression. Among these, vitamin D has attracted particular interest due to its well-established role in immune regulation and tumor suppression. Vitamin D, obtained through dietary intake or cutaneous synthesis via UV radiation, exerts its anticancer effects through the vitamin D receptor (VDR), which regulates cell proliferation, differentiation, and apoptosis [43]. Lower serum vitamin D levels have been associated with increased melanoma thickness, higher rates of metastasis, and poorer overall prognosis [43]. Some studies have suggested that vitamin D supplementation in individuals with deficient levels may reduce melanoma progression by enhancing immune surveillance and reducing chronic inflammation [44].

In addition to vitamin D, other vitamins and minerals, such as vitamin A, vitamin C, vitamin E, and selenium, have demonstrated potential protective effects against melanoma. Vitamin A, primarily obtained from animal liver, dairy products, and beta-carotene-rich vegetables, functions as a regulator of cell differentiation and apoptosis. Retinoids, a synthetic derivative of vitamin A, have shown promise in inhibiting melanoma cell proliferation and promoting apoptosis in vitro [32]. Similarly, vitamin C and E, potent antioxidants found in citrus fruits, nuts, and seeds, have been shown to reduce oxidative stress, enhance DNA repair, and promote immune function, collectively contributing to reduced melanoma risk [45]. Selenium, a trace mineral with antioxidant properties, has also been associated with reduced melanoma risk and improved overall immune function [46]. However, large-scale clinical trials are still needed to clarify the role of vitamin and mineral supplementation in melanoma prevention and management.

### 3.4. Alcohol and Caffeine

Epidemiological studies have identified a modest but statistically significant association between alcohol intake and increased melanoma risk. A pooled analysis of three large U.S. cohorts encompassing over 210,000 participants reported a 14% increase in risk of invasive melanoma per additional alcoholic drink consumed daily (hazard ratio [HR]: 1.14; 95% confidence interval [CI]: 1.00–1.29; *p*-trend = 0.04) [47]. Notably, this association was more pronounced for melanomas located on UV-protected sites, such as the trunk, suggesting mechanisms beyond ultraviolet UV exposure. Further, a 2018 meta-analysis encompassing 18 studies (12 case–control and 6 cohort) found a 29% increased melanoma risk among individuals with the highest alcohol consumption compared to those with the lowest [48]. Specific types of alcoholic beverages may differentially influence risk; for instance, white wine consumption has been associated with a higher melanoma risk, potentially due to its higher acetaldehyde content.

Biologically, alcohol metabolism produces acetaldehyde, a recognized carcinogen that can form DNA adducts, leading to mutations and impaired DNA repair mechanisms. Additionally, alcohol may act as a photosensitizer, enhancing skin sensitivity to UV radiation and promoting oxidative stress, thereby contributing to melanoma genesis [49]. However, it is important to consider potential confounding factors. Alcohol consumption often correlates with behaviors such as reduced sunscreen use and increased UV exposure, which themselves are established melanoma risk factors. Some studies have attempted to adjust for these variables, but residual confounding cannot be entirely ruled out. For example, a study noted that heavy drinkers might engage in riskier sun behaviors, potentially amplifying melanoma risk [50]. Conversely, caffeine consumption has been investigated for its potential protective effects against melanoma. Experimental studies have demonstrated that caffeine can enhance UV-induced apoptosis in keratinocytes, thereby facilitating the removal of damaged cells and reducing the likelihood of malignant transformation [51]. Caffeine’s inhibitory effect on the ATR-Chk1 pathway, a key regulator of DNA damage response, further contributes to its photoprotective properties [51]. Epidemiologically, higher caffeine intake has been associated with a reduced risk of basal cell carcinoma, a common skin cancer. While the direct evidence linking caffeine consumption to decreased melanoma risk is less robust, these findings suggest a potential protective role that warrants further investigation.

### 3.5. Dietary Patterns and Their Clinical Implications

There is growing recognition that overall dietary patterns, rather than isolated nutrients, may exert a more substantial influence on melanoma risk and progression. Among these, the Mediterranean diet has emerged as a potentially protective dietary model. This diet is typified by a high intake of fruits, vegetables, legumes, whole grains, nuts, and fish; moderate consumption of dairy and wine; and a predominant use of extra-virgin olive oil as the principal source of dietary fat. Operational definitions often include the consumption of ≥4 tablespoons of olive oil per day, ≥3 servings of legumes and fish per week, daily intake of fruits and vegetables, and limited intake of red and processed meats [52].

Epidemiological data have demonstrated an inverse association between adherence to the meditteranean diet and melanoma incidence, independent of established confounders such as ultraviolet exposure, skin phototype, and physical activity. Mechanistically, the meditteranean diet provides a high density of anti-inflammatory and antioxidant compounds, such as polyphenols, carotenoids, flavonoids, and omega-3 fatty acids, that mitigate oxidative stress, modulate pro-inflammatory cytokine signaling, and support immune surveillance, all of which are relevant to melanoma pathogenesis (Table 1) [53]. In addition to its potential in primary prevention, this diet may modulate the gut microbiota in a manner conducive to anti-tumor immunity. Diets rich in plant-derived fibers and polyphenols have been shown to promote microbial diversity and increase the abundance of SCFA-producing taxa, including *Faecalibacterium prausnitzii* and *Roseburia* spp., which play key roles in maintaining intestinal barrier integrity and regulating immune responses [54]. These microbial signatures have been associated with favorable responses to ICIs in melanoma and other solid tumors [54]. Conversely, Western dietary patterns, characterized by high intake of saturated fats, processed meats, and refined carbohydrates, are linked to increased systemic inflammation, microbial dysbiosis, and impaired immunotherapeutic efficacy. Collectively, current evidence supports the hypothesis that adherence to a Mediterranean dietary pattern may confer both preventative and therapeutic benefits in melanoma through mechanisms involving systemic inflammation, oxidative stress, and host–microbiome–immune interactions as summarized in Table 2. Further prospective studies and randomized controlled trials are warranted to validate these associations and to elucidate the impact of structured dietary interventions in melanoma prevention, progression, and treatment response.

### 3.6. Bioactive Compounds in Melanoma Prevention and Treatment

Dietary bioactive compounds, which include phytochemicals, polyphenols, and various plant-derived micronutrients, have garnered increasing attention for their potential role in preventing and managing melanoma. These compounds, found predominantly in fruits, vegetables, herbs, and certain beverages, exert anti-carcinogenic effects through their antioxidant, anti-inflammatory, and immunomodulatory properties. Among the most extensively studied compounds are curcumin, resveratrol, sulforaphane, and quercetin, each of which demonstrates promising therapeutic potential in melanoma prevention and treatment.

Curcumin, a polyphenolic compound derived from the rhizome of *Curcuma longa*, has been shown to exhibit significant anti-melanoma properties. Its primary mechanism of action involves the suppression of NF-κB signaling, which plays a central role in promoting inflammation, cell proliferation, and survival in melanoma cells [55]. Additionally, curcumin modulates oxidative stress by increasing superoxide dismutase (SOD) and glutathione peroxidase (GPx) activity, thereby reducing ROS production in melanoma cells. Preclinical studies have demonstrated that curcumin treatment can reduce melanoma cell viability, induce apoptosis, and suppress metastatic potential by inhibiting epithelial–mesenchymal transition (EMT) and angiogenesis [56]. Moreover, curcumin has been shown to enhance the efficacy of conventional therapies, including chemotherapy and immunotherapy, by increasing melanoma cell sensitivity to apoptotic signaling.

Resveratrol, a natural polyphenol found in grapes, red wine, and berries, has also demonstrated significant anti-melanoma effects. Resveratrol exerts its anticancer properties through several mechanisms, including the inhibition of cell proliferation, induction of apoptosis, and suppression of metastatic potential [57]. One of its primary mechanisms of action involves the activation of the Sirtuin 1 (SIRT1) pathway, which suppresses melanoma cell growth by inducing cell cycle arrest and promoting mitochondrial-mediated apoptosis [57]. Additionally, resveratrol downregulates pro-inflammatory cytokines, such as IL-6 and TNF-α, while concurrently reducing oxidative stress by enhancing antioxidant enzyme activity. Preclinical studies have further demonstrated that resveratrol can inhibit melanoma metastasis by reducing matrix metalloproteinase (MMP) activity, thus limiting extracellular matrix degradation and tumor cell migration [57].

Sulforaphane, a naturally occurring isothiocyanate derived from cruciferous vegetables such as broccoli, has demonstrated promising chemopreventive effects in melanoma. Sulforaphane’s primary mechanism of action involves the activation of the nuclear factor erythroid 2-related factor 2 (Nrf2) pathway, which enhances antioxidant enzyme expression and protects cells from oxidative stress-induced DNA damage [58]. Additionally, sulforaphane has been shown to inhibit the phosphatidylinositol 3-kinase (PI3K)/Akt signaling pathway, thereby reducing melanoma cell proliferation and promoting apoptosis [52]. Furthermore, sulforaphane suppresses the production of pro-inflammatory cytokines, such as IL-1β and IL-6, and has been demonstrated to potentiate the efficacy of immunotherapy by promoting an anti-tumor immune response [52].

Quercetin, a flavonoid widely found in fruits and vegetables such as onions, apples, and berries, has also demonstrated anti-melanoma properties. Quercetin’s primary anticancer mechanism involves the inhibition of the mitogen-activated protein kinase (MAPK) and PI3K/Akt signaling pathways, both of which are critical for melanoma cells’ survival, proliferation, and invasion [53]. Additionally, quercetin exerts antioxidant effects by scavenging free radicals and enhancing the expression of antioxidant enzymes, thereby reducing oxidative stress in melanoma cells. Quercetin has also been shown to induce cell cycle arrest in melanoma cells by upregulating p53 and p21 expression while downregulating cyclin-dependent kinases (CDKs) [53]. Moreover, quercetin can potentiate the effects of chemotherapy by increasing drug accumulation in melanoma cells and sensitizing them to apoptosis. Collectively, these bioactive compounds demonstrate promising therapeutic potential in melanoma prevention and treatment. Their ability to modulate oxidative stress, suppress pro-inflammatory signaling, and enhance apoptotic responses highlights their potential as adjunctive therapies in melanoma management. However, further clinical trials are needed to validate their long-term efficacy and safety in melanoma patients and to elucidate their optimal dosages for therapeutic use.

## 4. Limitations and Future Directions

Despite increasing interest in the influence of diet on melanoma prevention, progression, and therapeutic response, substantial gaps in the evidence base persist. Much of the current literature is derived from observational studies, which, while instrumental in generating hypotheses about dietary patterns and melanoma risk, are inherently limited by confounding factors, recall bias, and the absence of randomization. Mechanistic insights gleaned from preclinical and animal models, though valuable, often lack direct translatability to human populations. The paucity of randomized controlled trials further limits the ability to draw causal inferences, thereby impeding the formulation of evidence-based dietary guidelines. Moreover, variability in study designs, dietary assessment methodologies, and population characteristics hinders cross-study comparability and generalizability. Interactions between diet and other key determinants of melanoma risk, such as UV radiation exposure, physical activity, and genetic susceptibility, are frequently underexplored. Notably, the influence of diet on melanoma recurrence and long-term survivorship remains an understudied area, leaving clinicians with limited guidance for dietary counseling in this population. The emerging data underscore a complex interplay between diet, the gut microbiome, and melanoma outcomes. Dietary components such as fiber significantly modulate the composition and function of the gut microbiota, with downstream effects on host immune responses. Non-digestible carbohydrates, including inulin, fructans, and galactooligosaccharides, serve as fermentable substrates for commensal bacteria such as Bifidobacterium, Lactobacillus, and Roseburia. These taxa contribute to intestinal homeostasis through the production of SCFAs, particularly butyrate. Butyrate has well-established anti-inflammatory and anti-neoplastic properties, acting as a histone deacetylase inhibitor to promote histone acetylation, induce apoptosis, and inhibit tumor cell proliferation.

In oncologic contexts, SCFA-mediated modulation of the tumor microenvironment appears to enhance mucosal immunity and may potentiate the efficacy of ICIs. Increased fecal butyrate levels following a high fiber intake suggest cooperative interactions among the microbial taxa involved in SCFA biosynthesis. Clinically, high dietary fiber consumption has been associated with improved progression-free survival in melanoma patients undergoing anti-PD-1 therapy. This benefit has been linked to increased gut microbial diversity and the enrichment of immunostimulatory taxa such as Ruminococcaceae and Faecalibacterium. Preclinical studies support a microbiota-dependent mechanism, as these effects are diminished in germ-free models. The restoration of anti-PD-1 efficacy has been achieved through microbial recolonization with taxa such as Akkermansia muciniphila and Enterococcus hirae, both of which have demonstrated cross-model relevance in melanoma and lung carcinoma. Additionally, commensals like Alistipes indistinctus, found to be enriched in ICI responders, have further enhanced therapeutic responses in experimental models. While most research to date has centered on primary prevention, there is a critical need to investigate how dietary factors influence melanoma recurrence, metastasis, and long-term survival. Precision nutrition strategies—tailored to individual microbiome profiles and metabolic phenotypes—may offer a promising adjunct to existing treatment paradigms. Robust longitudinal cohort studies and randomized dietary intervention trials are urgently needed to establish actionable, evidence-based dietary recommendations for melanoma survivorship and immunotherapy optimization.

## 5. Conclusions

The influence of diet on melanoma prevention, progression, and treatment outcomes represents a promising yet underexplored area of research. Accumulating evidence suggests that specific dietary components, including bioactive compounds, antioxidants, and micronutrients, can modulate oxidative stress, inflammation, and immune responses, thereby influencing melanoma pathogenesis. Additionally, emerging data highlighting the intricate relationship between diet, gut microbiome composition, and immune function further emphasize the potential role of dietary interventions in enhancing melanoma treatment efficacy and improving patient outcomes. While current epidemiological and experimental studies provide compelling evidence supporting the role of diet in melanoma prevention, significant gaps in the knowledge remain regarding the direct impact of dietary patterns on melanoma progression, survivorship, and treatment response. The lack of large-scale studies limits the ability to establish definitive dietary recommendations for melanoma patients. Additionally, the complex interplay between dietary factors, gut microbiota, and immune response necessitates further investigation to identify optimal dietary strategies that may enhance immunotherapy efficacy and reduce melanoma recurrence. Moving forward, future research efforts should prioritize conducting well-designed clinical trials to assess the therapeutic potential of dietary interventions in melanoma prevention and management. Additionally, exploring the role of the gut microbiome in modulating melanoma progression and treatment response may uncover novel targets for dietary modulation. As the field of personalized medicine continues to evolve, integrating individualized dietary recommendations based on a patient’s gut microbiome composition, metabolic profile, and treatment regimen may optimize melanoma outcomes. Ultimately, leveraging the power of dietary modification as an adjunctive strategy in melanoma management holds significant promise in reducing melanoma burden and improving long-term patient survival.

## Figures and Tables

**Table 1:** Key dietary components and mechanisms in melanoma management.

Stage	Goal	Intervention	Details/Targets	Supporting Evidence
Prevention	Reduce incidence in high-risk individuals	Mediterranean Diet	≥4 tbsp/day extra-virgin olive oil. ≥5 servings/day fruits and vegetables. 2–3 servings/week fatty fish. <1 serving/week red/processed meat.	Observational studies, including the EPIC cohort, have associated adherence to the Mediterranean diet with a reduced risk of various cancers, including melanoma. The diet’s high content of antioxidants and omega-3 fatty acids may mitigate oxidative stress and inflammation.
		Antioxidant-Rich Foods	Incorporate foods high in vitamins C and E, beta-carotene, and selenium. Examples: citrus fruits, nuts, leafy greens, and whole grains.	Antioxidants may protect skin cells from UV-induced damage by neutralizing free radicals, thereby potentially reducing melanoma risk.
Active Treatment	Enhance response to immunotherapy and reduce treatment-related toxicity	Dietary Fiber Intake	≥30 g/day from whole grains, legumes, fruits, and vegetables.	Higher dietary fiber intake has been linked to improved gut microbiota diversity, which may enhance the efficacy of immune checkpoint inhibitors in melanoma treatment.
		Probiotics/Prebiotics	Emphasize intake of prebiotic foods (e.g., garlic, onions, bananas) and probiotic-rich foods (e.g., yogurt, kefir). Target beneficial bacteria such as *Faecalibacterium prausnitzii* and *Bifidobacterium longum*.	Modulating the gut microbiome through diet may improve immune responses and treatment outcomes in melanoma patients.
		Vitamin D Supplementation	Screen for deficiency and supplement accordingly. Aim for serum 25(OH)D levels within optimal range.	Adequate vitamin D levels may support immune function and have been associated with better outcomes in melanoma patients undergoing immunotherapy.
Survivorship	Reduce recurrence and support long-term immune health	Anti-Inflammatory Diet	Emphasize Mediterranean or plant-forward diets rich in phytonutrients and omega-3 fatty acids<br>- Limit intake of processed and red meats, refined sugars, and saturated fats.	Anti-inflammatory diets may reduce systemic inflammation, support immune function, and lower the risk of melanoma recurrence.
		Nutritional Monitoring	Regular consultations with a registered dietitian<br>- Personalized nutrition plans based on individual needs and treatment side effects.	Ongoing nutritional support can help manage treatment-related side effects, maintain optimal nutritional status, and improve quality of life in melanoma survivors.

**Table 2:** Protective and dietary factors and dietary risk factors in melanoma.

Factor	Association	Evidence Tier	Supporting Evidence
Mediterranean Diet	Protective	High	Adherence to the Mediterranean diet has been associated with a reduced risk of melanoma, potentially due to its high content of antioxidants and anti-inflammatory nutrients.
Dietary Fiber Intake	Protective	Moderate	Higher fiber intake may improve gut microbiota diversity, enhancing immune responses and the efficacy of immunotherapy in melanoma patients.
Antioxidant-Rich Foods	Protective	Moderate	Consumption of foods rich in antioxidants may protect against UV-induced skin damage, thereby reducing melanoma risk.
Alcohol Consumption	Risk	Moderate	Some studies suggest a modest association between alcohol intake and increased melanoma risk, possibly due to alcohol-induced immunosuppression and increased skin photosensitivity.
Western Dietary Pattern	Risk	Moderate	Diets high in red and processed meats, refined sugars, and saturated fats have been linked to increased systemic inflammation and may elevate melanoma risk.
